# No drugs, more sex? And Rock’n Roll: effective non-operative treatments and practical management strategies for older adults with lumbar spinal stenosis

**DOI:** 10.1186/s12998-025-00590-3

**Published:** 2025-07-21

**Authors:** Carlo Ammendolia

**Affiliations:** 1 Department of Surgery, University of Toronto, Toronto, Canada; 2 Sinai Health, Toronto, Canada

**Keywords:** Lumbar spinal stenosis, Neurogenic claudication, Non-operative, Management, Narrative review, Sexual dysfunction, Evidence summary

## Abstract

Lumbar spinal stenosis is a growing problem among older adults, associated with significant disability and socio-economic burden. Neurogenic claudication is the most common clinical syndrome caused by LSS with pain being the predominant symptom and limited walking the main impairment. Lumbar spinal stenosis can also impact sexual function in older adults, necessitating greater awareness of this association. Pain and impaired function can lead to psychosocial distress, including hopelessness, anxiety, and isolation, further compounding disability. Recent clinical practice guidelines recommend non-operative treatment as the first-line approach, including manual therapy, exercise, and education. However, these guidelines lack details on the practical application of these interventions in clinical practice. This narrative review explores the epidemiology of lumbar spinal stenosis, the evidence supporting non-operative care, and practical management strategies. It also highlights the relationship between sexual dysfunction and lumbar spinal stenosis.

## Introduction

Lumbar spinal stenosis (LSS) is a leading cause of pain, disability, and loss of independence in older adults [1]. It is usually caused by age-related degenerative changes or osteoarthritis of the lumbar spine [2]. These degenerative changes include thinning and bulging intervertebral discs, hypertrophy of the facet joints, and in-folding of the ligamentous flavum leading to structural narrowing of the central and/or lateral spinal canals [2]. Neurogenic claudication, characterized by buttock and/or leg pain, either unilaterally or bilaterally, and/or numbness, tingling, weakness, or heaviness of the leg(s), that is worse with standing and/or walking and is better with sitting and stooping forward, is the hallmark clinical syndrome of LSS [3]. Neurogenic claudication is a clinical diagnosis and therefore imaging is not necessary to make this diagnosis [4].

Pain is the most prevalent symptom of LSS, and limited walking is the most common impairment [5, 6]. People with LSS experience greater walking limitations than people with hip or knee osteoarthritis [7] and are more functionally impaired than individuals with systemic lupus erythematosus, chronic obstructive lung disease, and congestive heart disease [1]. Moreover, LSS can also impact urogenital function leading to urinary and sexual dysfunction [8]. There is a call to action for increased awareness on the association of lumbar spinal stenosis and other spinal disorders on sexual function [8, 9].

Reduced mobility and overall functional decline can lead to feelings of hopelessness, depression, and isolation, further exacerbating LSS-related disability [10]. Although LSS is the most common indication for spine surgery in people older than 65, most people who seek care for LSS receive non-operative care [6] with a cost of billions of dollars each year [11]. However, the effectiveness of most non-operative treatments is unknown [12]. This review aims to summarize the epidemiology and evidence for effective non-operative treatments for LSS, explore the association with sexual dysfunction, and provide practical management strategies.

## Methods

This is a narrative review based primarily on recent clinical practice guidelines, systematic reviews, and clinical trials relevant to LSS. A formal search strategy or quality assessment of the evidence was not conducted. The breadth of the topic precluded a formal systematic review.

### Prevalence

Globally, approximately 103 million individuals suffer from LSS [13], representing 11% of the general adult population and 39% of clinical populations, with the prevalence increasing with age [14, 15]. The prevalence of LSS is growing rapidly in most western countries due to the increase in the number and proportion of people over the age of 65 [16]. Although imaging reveals a higher LSS severity in men [17], symptom severity is similar between the sexes [18].

### Natural history

The natural history of LSS is variable, with symptoms fluctuating over time and most individuals rarely experiencing a rapid decline in symptoms. About a third of individuals with mild-moderate LSS experience gradual worsening symptoms, a third will remain stable, and one third improve [19, 20]. There are no known reliable predictors of worsening disability in LSS; however, in general, current functional status predicts future functional status [6]. For example, frailty among people with LSS leads to worse functional outcomes.

### Etiology and pathophysiology

Neurogenic claudication is the most common symptom of LSS and is thought to result from ischemia of the nerve roots [21]. Narrowed spinal canals lead to venous congestion and backup of the cerebral spinal fluid restricting blood flow to the nerve roots [21]. Lumbar flexion and sitting increases spinal canal volume, decreases venous congestion, improve blood flow to the nerve roots, and decreases symptoms [21]. Interventions that increase spinal canal volume, such as increasing lumbar intersegmental flexion or reducing lumbar lordosis while standing and walking, may reduce symptoms [22].

### Diagnosis

There are no agreed-upon diagnostic criteria for neurogenic claudication; however, key features of the history and physical examination are usually sufficient to make the clinical diagnosis [3]. These features include older age, lower extremity symptoms worse with walking, standing, and lumbar extension, and relieved with flexion postures such as stooping forward and/or sitting. Other diagnostic features include worsening balance and urinary disturbances. There are many imposters of LSS [62]. Many older adults have concurrent comorbid conditions that can also impact lower extremity symptoms and walking ability, leading to diagnostic uncertainties [62]. Peripheral vascular disease [23, 24], degenerative hip disease [25, 26], greater trochanteric syndrome [27], cervical myelopathy [62], and diabetic neuropathy are all common conditions that can simulate or coexist with LSS [62]. Accurate diagnosis is imperative to provide appropriate and timely treatment.

### Imaging

Once red flags are ruled out at the initial assessment, imaging is of little value from a non-operative perspective [14]. Imaging findings often do not correlate with patients’ symptoms and are rarely useful for predicting outcomes [19]. Neurogenic claudication is a clinical diagnosis that describes a clinical pattern of symptoms and therefore imaging is not necessary [22]. Imaging has a role when patients fail or are unsatisfied with conservative treatment and are considering surgical intervention or have worsening neurological deficits [19].

### Psychosocial factors

The psychosocial impact of LSS cannot be underestimated [10]. Hopelessness, despair, anxiety, depression, and isolation are often associated with LSS and can significantly exacerbate physical and functional disability. Walking limitations lead to a shrinking world and lost aspirations for active and fulfilling retirement years. Fear of movement and the future, poor self-efficacy, low motivation, negative expectations, and depressed moods are factors that can impact outcomes in LSS. Effective treatment should consider the physical, functional, and psychosocial aspects of LSS [22].

### Sexual dysfunction

Research on the impact of LSS on sexual function is very limited. Neural ischemia of the cauda equina can affect the functioning of the preganglionic fibers through S2-4 nerve roots [8]. These nerve fibers eventually synapse in the pelvic plexus, and the post-ganglionic nerve fibers then innervate the bladder and sexual structures. Lack of nerve supply and axonal transport blockage can give rise to urinary and sexual dysfunction in individuals with LSS [33]. Over a dozen case reports have highlighted intermittent neurogenic priapism while walking in patients with LSS [29–32] and other very low-quality studies have reported erectile dysfunction secondary to LSS [33, 34]. One retrospective study suggests that the prevalence of erectile dysfunction alongside LSS was 89.5% among patients scheduled for decompression surgery [28 L]. Due to very limited published data, no conclusions can be made on the epidemiology of LSS-related sexual dysfunction. This is an area that needs further research and awareness [8].

### Management

Recent clinical practice guidelines recommend multi-modal conservative care as first-line treatment for LSS incorporating manual therapy, supervised exercise, and education delivered through a cognitive-behavioral approach [14, 35]. Below is a summary of evidence and practical strategies for non-operative care.

### Oral medications

Oral medications are commonly prescribed for the treatment of LSS [36]. However, due to the lack of adequate evidence for their efficacy and the potential for significant side effects, especially in older adults, they are not recommended as first-line treatment [12, 14, 35].

#### Antiepileptic agents

Pregabalin and gabapentin are widely prescribed for LSS. While effective for some types of neuropathic pain, several low quality randomized controlled trials (RCTs) have shown no benefit for LSS and an increased risk of adverse effects including dizziness and drowsiness [37–40]. Current guidelines and systematic reviews do not recommend these agents for LSS or back-related lower extremity symptoms [12, 14, 35, 41–43].

#### Other medications

Other commonly prescribed medications for LSS include non-steroidal anti-inflammatory drugs (NSAIDs), acetaminophen, and opioids. There is low-quality evidence showing opioids are no better than placebo and there are no placebo-controlled trials examining NSAIDs or acetaminophen for LSS [12]. Neurogenic claudication symptoms are due to neuro-ischemia, which is not consistent with an inflammatory process. High-quality studies conclude that acetaminophen is not effective for back or back-related leg symptoms [44]. Significant adverse effects of opioids and NSAIDs combined with the lack of evidence for their effectiveness, suggest these drugs should not be recommended for LSS.

### Epidural injections

Approximately 25% of all epidural injections are performed for LSS, despite the lack of supporting evidence [12, 45]. Moderate evidence from RCTs shows no clinically important short or long-term benefit from these injections which typically contain steroids and lidocaine [12, 14, 35, 46]. The neuro-ischemic nature of neurogenic claudication suggests these formulations would not provide long-term benefit.

### Acupuncture

Conflicting and low-quality evidence does not support acupuncture as first line treatment for LSS [12, 14, 35, 47].

### Spinal manipulation

Low quality evidence demonstrated no benefit for spinal manipulation as a standalone treatment for LSS [14, 35, 48]. There is one case study demonstrating improvement of sexual dysfunction after spinal manipulation in an older man with LSS, but this is preliminary evidence, and no conclusions can be made about the effectiveness of this treatment for sexual dysfunction in LSS [49].

### Surgery

There is conflicting and low-quality evidence comparing surgery to conservative care for LSS [12, 50]. Overall evidence suggests that surgery is effective for leg-dominant symptoms, but the benefits diminish over time [51]. In the absence of red flags for serious pathology or severe debilitating symptoms, a course of non-surgical treatment should be first-line treatment for LSS [14, 35].

### Multi-modal conservative care

Moderate evidence supports a multi-modal approach incorporating manual therapy, exercise, and education using a cognitive-behavioral approach as first-line treatment for LSS [35]. RCTs have demonstrated short and long-term improvements in pain, walking distance, and overall function with this approach [22, 52–54].

### Practical application of a comprehensive multimodal conservative care for LSS

Although clinical practice guidelines recommend multi-modal care for LSS, there is a lack of details or practical strategies for delivering this care. Below are details on implementing a comprehensive multimodal conservative care program based on the 6-week Boot Camp Program for LSS [12, 22, 52].

This program is delivered using a biopsychosocial model of care, focusing on self-management and addressing the physical aspects of LSS, including pain and limited spinal mobility, functional impairments such as limited walking and standing ability, and psychosocial barriers (fear, low self-efficacy, and mood).

**Fig. 1:**
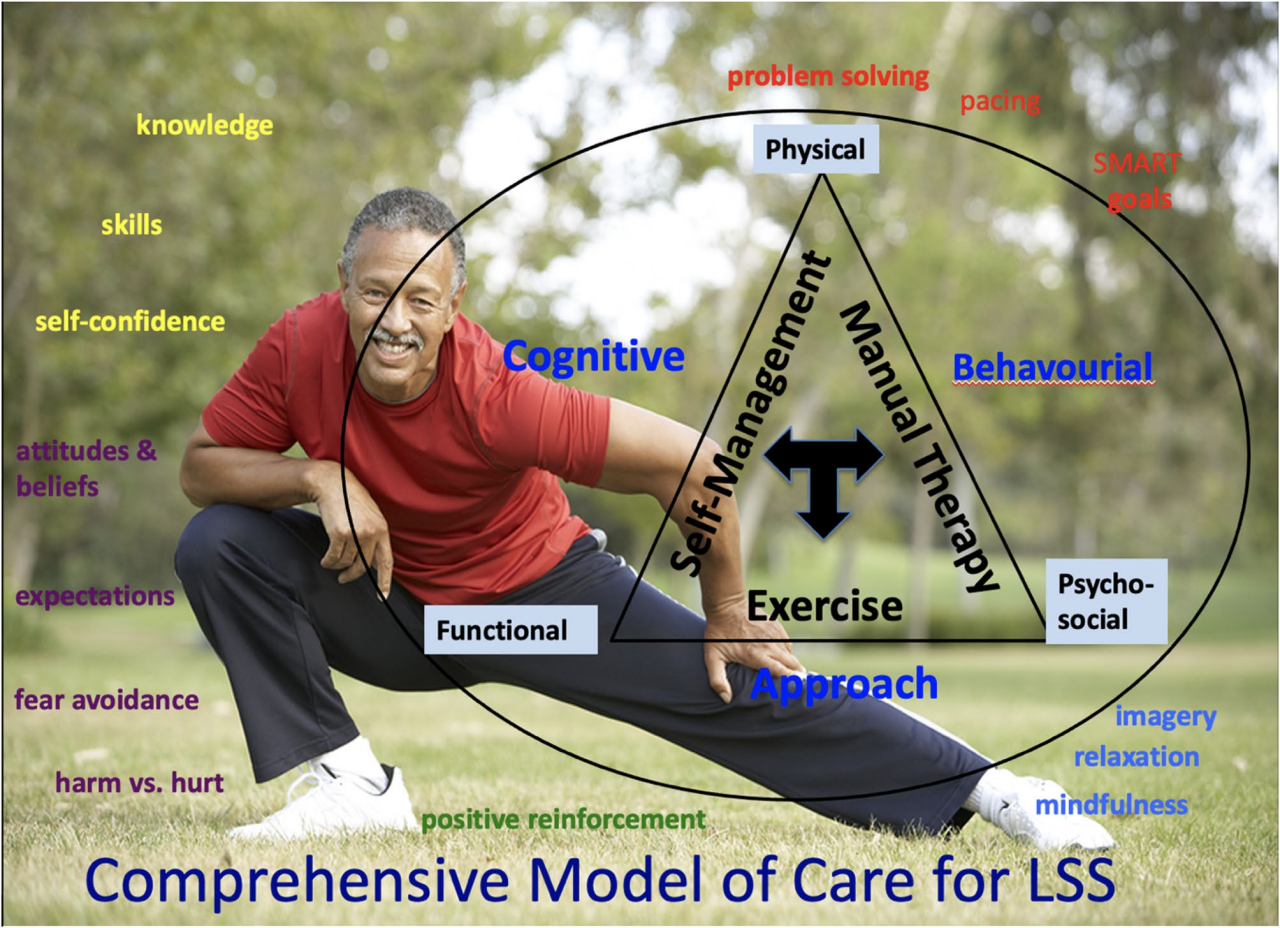
Conceptual framework for comprehensive multimodal conservative care for LSS.

- **Pre-program screening and outcome measures**.

Before starting the program, patients should be screened for potential psychosocial barriers or yellow flags, such as fear of movement, low self-efficacy, negative or unrealistic expectations, low motivation, and mood [55, 56]. Tailored interventions should address any identified psychosocial barriers and integrated with interventions for physical and functional impairments. Baseline pain (average weekly VAS pain score) and a non-stop walk test (using a mobile phone app) to assess the maximum number of steps should be obtained and recorded weekly during the six-week program. Patient-centered realistic functional goals should be discussed, emphasizing that complete resolution of symptoms is unlikely, and the focus is on maximizing function in both the short and long term. Currently, there is no agreed upon core set of outcome measures for LSS and most measures used to assess outcomes in LSS have inadequate or unknown measurement properties [57]. A core set should include outcomes that are valid, reliable, responsive and relevant to patients and clinicians and should include both self-report and objective measures [57].

- **Program Delivery**.

- Manual Therapy.

Manual therapy is delivered at each visit of the six-week program. The goal is to improve intersegmental lumbar spine flexion using mobilization and/or manipulation, stretch and relax tight muscles that facilitate lumbar extension (erector spinae and psoas muscles), and perform nerve flossing techniques to improve mobility and blood flow to the sciatic and/or femoral nerves [22].

(b) Exercise.

Specific exercises are introduced at each visit of the program and become part of a structured home program that is recommended for life. During the six-week program, exercises are performed twice per day and then reduced to once per day at the end of the program. Exercises aim to improve cardiovascular fitness, lower extremity strength, spinal and hip flexibility and core strengthening.

(c) Education using a cognitive behavioral approach

At each visit, tailored education is provided to address identified psychosocial barriers and offer information on LSS, its underlying causes, prognosis, and strategies for problem-solving and pacing. The goal is to provide knowledge, skills, and self-confidence for life-long self-management. Positive reinforcement and feedback from weekly pain scores, step counts, and functional goals can help to build self-confidence, reduce fear and improve mood. Explanations about hurt vs. harm pain, working through the pain, and mitigating negative expectations and maladaptive attitudes and beliefs are provided to high-risk patients.

**Table 1:** Practical strategies aimed at psychosocial barriers

Psychosocial Barriers	Interventions– aimed at changing attitudes/beliefs, building self-confidence, skills and knowledge	ROF	W1	W2	W3	W4	W5	W6
**Negative Expectations** “I will not get better” “This treatment will not help me” “ I will get worse”	**Validate Concerns** “yes I understand you are concerned”. Show **empathy and compassion** Provide **positive expectations** Use research findings/ data, e.g. 85% significantly improve function end of program **Not about eliminating pain** about maximizing function. But on average pain decreases significantly Provide **positive reinforcement**, encouragement and **positive messaging and feedback**. Identify positive changes each visit.	x	x	x	x	x	x	x
**Pain/fear Avoidance Behaviour** “Activity will cause more pain” “I am afraid to do things” “Activity hurts me”	**Validate Concerns** “yes I understand you are concerned”. Show **empathy and compassion** Explain **harm vs. hurt** pain. Pain does not mean damage **Ok to feel some pain** with activity/walking. 60% are worse before better with program. **Activity is key** to long term benefit Show how to **problem solve**- tips on self-management (e.g. use pelvic tilt). Demonstrate **pacing**/ use heat/ice Complete **goal setting exercise** SMART goals. Use **imagery exercises** to reduce pain and fear	x	x	x	x	x	x	x
**Low/depressed mood/isolation** “I cannot cope” “I feel hopeless” “I want to stay home”	**Validate Concerns** “yes I understand” Show **empathy and compassion** Provide **positive reinforcement**/**expectations** and **build self-confidence.** Identify positive changes each visit. Encourage **pacing**,** socialization** and use **goal setting exercise** and have a plan Use **imagery exercises** to reduce pain and fear and improve mood	x	x	x	x	x	x	x

ROF = report of findings or pre-treatment consultation. W = week

Strategies like imagery, relaxation, and mindfulness are taught and aimed at reducing fear and anxiety, especially when planning functional activities. Patients are also instructed on how to use the pelvic tilt while standing and walking. The pelvic tilt aims to reduce the lumbar lordosis and increase intersegmental flexion, improve blood flow to the spinal nerves, and enhance walking and standing tolerance [22].

- **Contextual Factors in Program Delivery**.

Contextual factors are external and internal factors beyond the treatment that can have a profound impact on outcomes [58, 59]. These effects are also known as placebo and nocebo effects. It is estimated that contextual factors can have outcomes similar in magnitude to treatment effects [58]. Examples include the patient-practitioner interactions, such as validating patients’ concerns, showing empathy and compassion and using positive language. There are also patient and practitioner-specific factors, treatment, and environmental factors. We have developed a conceptual framework on how to enhance positive contextual factors to improve outcomes [60].

**Fig. 2:**
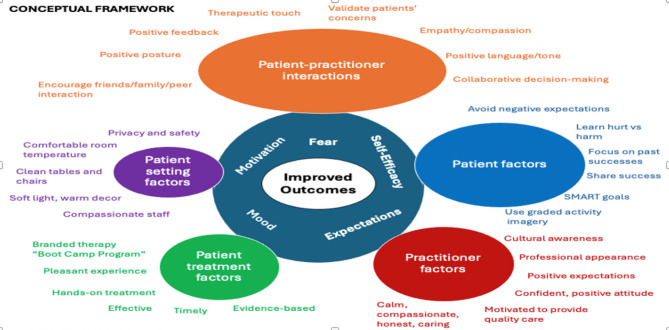
Conceptual framework to enhance positive contextual factors.

Using these strategies to enhance contextual factors while delivering care for LSS may improve outcomes, especially among high-risk patients who present with psychosocial barriers to recovery.

### Management of sexual dysfunction in LSS

There is very sparse published literature on the management of sexual dysfunction in LSS. There is one case study demonstrating improvement of sexual dysfunction after spinal manipulation in an older man with LSS [49]. In a recent prospective study, patients treated with rehabilitative exercises and mobility and fascial stretches, had significantly better sexual satisfaction scores at 12 months compared to patients treated surgically for LSS [61]. In another recent surgical study, patients with lumbar or cervical spinal stenosis who underwent decompression surgery did not have any improvement is erectile dysfunction [34]. Given the very low quantity and quality of the current evidence, no conclusions can be made about the effectiveness of any intervention for sexual dysfunction in LSS [49].

## Conclusions

First-line treatment for LSS causing neurogenic claudication should be conservative without oral drugs or injections (no drugs) and should include structured manual therapy, supervised exercise, and education delivered using a cognitive-behavioral approach with the enhancement of positive contextual factors. This comprehensive approach will likely lead to improved functional outcomes in older adults (Rock’n Roll). More research is needed on the cause and effect of LSS on sexual dysfunction in older adults and the potential for effective conservative interventions (more sex??).

## Data Availability

No datasets were generated or analysed during the current study.
