# Correction to: No drugs, more sex? And Rock’n Roll: effective non-operative treatments and practical management strategies for older adults with lumbar spinal stenosis

**DOI:** 10.1186/s12998-026-00637-z

**Published:** 2026-04-08

**Authors:** Carlo Ammendolia

**Affiliations:** 1 Department of Surgery, University of Toronto, Toronto, Canada; 2 Sinai Health, Toronto, Canada

**Correction to: Chiropr Man Therap 33, 27 (2025).** 10.1186/s12998-025-00590-3

The author apologizes to the readers for errors in the reference list of the original publication. ChatGPT was used to assist with formatting references in Vancouver style, which led to the inclusion of fictitious references that the author failed to verify prior to submission. The article content & conclusions remain unchanged.

Furthermore, there were several citation errors:

- Citation [22] in the ‘Diagnosis’ section should have cited a different reference which was not originally included. The citations for reference [22] in the ‘Diagnosis’ section have been changed to reference [62], which has now been added.
- Citation [5] in the ‘Diagnosis’ section should have cited reference [3], which was present in the original publication.
- Citation [28] in the ‘Sexual Dysfunction’ section should have cited reference [33], which was present in the original publication.

The incorrect and correct references are shown in this correction article, the original article has been updated.

**Table:** 

Correct references	Incorrect references
1. Fanuele JC, Birkmeyer NJ, Abdu WA, Tosteson TD, Weinstein JN. The impact of spinal problems on the health status of patients: have we underestimated the effect? Spine (Phila Pa 1976). 2000 Jun 15;25(12):1509-14	1. Fanuele JC, Abdu WA, Hanscom B, Weinstein JN. Association between obesity and functional status in patients with lumbar spinal stenosis. Spine (Phila Pa 1976). 2000;25(18):2133–8.
2. Katz JN, Zimmerman ZE, Mass H, Makhni MC. Diagnosis and management of lumbar spinal stenosis: a review. Jama. 2022 May 3;327(17):1688-99	2. Takahashi K, Miyazaki T, Takino T, Matsui T, Tomita K. Epidural pressure measurements. Relationship between epidural pressure and posture in patients with lumbar spinal stenosis. Spine (Phila Pa 1976). 1995;20(6):650–3.
3. Suri P, Rainville J, Kalichman L, Katz JN. Does this older adult with lower extremity pain have the clinical syndrome of lumbar spinal stenosis? Jama. 2010 Dec 15;304(23):2628-36.	3. Katz JN, Zimmerman ZE, Mass H, Makhni MC. Diagnosis and management of lumbar spinal stenosis: A review. JAMA. 2022;327(17):1688–99.
4. Deer T, Sayed D, Michels J, Josephson Y, Li S, Calodney AK. A review of lumbar spinal stenosis with intermittent neurogenic claudication: disease and diagnosis. Pain Med. 2019 Dec 1;20(Supplement_2):S32-44.	4. Chou R, Baisden J, Carragee EJ, Resnick DK, Shaffer WO, Loeser JD. Surgery for low back pain: a review of the evidence for an American pain society clinical practice guideline. Spine (Phila Pa 1976). 2009;34(10):1094–109.
5. Houle M, Bonneau JD, Marchand AA, Descarreaux M. Physical and psychological factors associated with walking capacity in patients with lumbar spinal stenosis with neurogenic claudication: a systematic scoping review. Front Neurol. 2021 Sep 9;12:720662.	5. Suri P, Rainville J, Kalichman L, Katz JN. Does this older adult with lower extremity pain have the clinical syndrome of lumbar spinal stenosis? JAMA. 2010;304(23):2628–36.
6. Lurie J, Tomkins-Lane C. Management of lumbar spinal stenosis. BMJ. 2016 Jan 4;352.	6. Lurie J, Tomkins-Lane C. Management of lumbar spinal stenosis. BMJ. 2016;352:h6234.
7. Winter CC, Brandes M, Müller C, Schubert T, Ringling M, Hillmann A, Rosenbaum D, Schulte TL. Walking ability during daily life in patients with osteoarthritis of the knee or the hip and lumbar spinal stenosis: a cross-sectional study. BMC Musculoskelet Disord. 2010 Oct 12;11(1):233.	7. Winter CC, Brandes M, Müller C, Schubert T, Ringling M, Hillmann A, et al. Walking ability during daily life in patients with osteoarthritis of the hip or knee. J Orthop Res. 2010;28(4):490–7.
8. Gandhi J, Shah J, Joshi G, Vatsia S, DiMatteo A, Joshi G, Smith NL, Khan SA. Neuro-urological sequelae of lumbar spinal stenosis. Int J Neurosci. 2018 Jun 3;128(6):554 − 62	8. Gandhi J, Healy M, Smith N, Patel S, Joshi G, Khan SA. Implications of urogenital pain in the context of lumbar spinal stenosis: an integrative review. Int J Neurosci. 2017;127(12):1110–8.
9. Goldstein SW, Trofimenko V, Winter AG, Rubin RS, Komisaruk BR, Kissee J, Kim CW, Goldstein I. 188 Lumbar and Sacral Spinal-Mediated Neurogenic Sexual Dysfunction: Pathophysiology, Diagnosis and Treatment. J Sex Med. 2018 Feb;15(Supplement_1):S59-.	9. Gandhi J, Dagur G, Warren K, Smith NL, Sheynkin YR, Zumbo A, et al. The role of degenerative spinal pathology in sexual dysfunction: a narrative review. Int J Impot Res. 2017;29(6):219–27.
10. Ammendolia C, Schneider M, Williams K, Zickmund S, Hamm M, Stuber K, Tomkins-Lane C, Rampersaud YR. The physical and psychological impact of neurogenic claudication: the patients’ perspectives. J Can Chiropr Assoc. 2017 Mar;61(1):18.	10. Ammendolia C, Schneider M, Williams K, Zickmund S, Hamm M, Stuber K, et al. The physical and psychological impact of neurogenic claudication: the patients’ perspectives. J Can Chiropr Assoc. 2017;61(1):18–31.
11. Tosteson AN, Lurie JD, Tosteson TD, Skinner JS, Herkowitz H, Albert T, Boden SD, Bridwell K, Longley M, Andersson GB, Blood EA. Surgical treatment of spinal stenosis with and without degenerative spondylolisthesis: cost-effectiveness after 2 years. Ann Intern Med 2008 Dec 16;149(12):845 − 53.	11. Tosteson AN, Lurie JD, Tosteson TD, Skinner JS, Herkowitz H, Albert T, et al. Surgical treatment of spinal stenosis with and without degenerative spondylolisthesis: cost-effectiveness after 2 years. Ann Intern Med. 2008;149(12):845–53.
12. Ammendolia C, Hofkirchner C, Plener J, Bussières A, Schneider MJ, Young JJ, Furlan AD, Stuber K, Ahmed A, Cancelliere C, Adeboyejo A. Non-operative treatment for lumbar spinal stenosis with neurogenic claudication: an updated systematic review. BMJ open. 2022 Jan 1;12(1):e057724.	12. Ammendolia C, Côté P, Rampersaud YR, Southerst D, Schneider M, Budgell B, et al. The boot camp program for lumbar spinal stenosis: a protocol for a randomized controlled trial. Chiropr Man Th. 2018;26:1.
13. Ravindra VM, Senglaub SS, Rattani A, Dewan MC, Härtl R, Bisson E, Park KB, Shrime MG. Degenerative lumbar spine disease: estimating global incidence and worldwide volume. Global Spine J. 2018 Dec;8(8):784 − 94.	13. Ravindra VM, Senglaub SS, Rattani A, Dewan MC, Hartl R, Bisson E, et al. Degenerative lumbar spine disease: estimating global incidence and worldwide volume. Global Spine J. 2018;8(8):784–94.
14. Jensen RK, Harhangi BS, Huygen F, Koes B. Lumbar spinal stenosis. BMJ. 2021 Jun 29;373.	14. Jensen RK, Jensen TS, Koes B, Pedersen H, Schiøttz-Christensen B. Prevalence of lumbar spinal stenosis in general and clinical populations: a systematic review and meta-analysis. Eur Spine J. 2020;29(9):2143–63.
15. Bagley C, MacAllister M, Dosselman L, Moreno J, Aoun SG, El Ahmadieh TY. Current concepts and recent advances in understanding and managing lumbar spine stenosis. F1000Res. 2019 Jan 31;8:F1000 Faculty Rev-137.	15. Bagley C, Gokaslan ZL, Bydon M. Lumbar spinal stenosis: contemporary medical management. J Neurosurg Sci. 2019;63(3):227–35.
16. Maeda T, Hashizume H, Yoshimura N, Oka H, Ishimoto Y, Nagata K, Takami M, Tsutsui S, Iwasaki H, Minamide A, Nakagawa Y, Yukawa Y, Muraki S, Tanaka S, Yamada H, Yoshida M. Factors associated with lumbar spinal stenosis in a large-scale, population-based cohort: The Wakayama Spine Study. PLoS One. 2018 Jul 18;13(7):e0200208.	16. Cheng X, Zhang L, Zhang K, Zhang G, Hu Y, Zhang L, et al. Prevalence of lumbar spinal stenosis in the elderly population in china: a cross-sectional study. PLoS Med. 2020;17(6):e1003092.
17. Travis Caton M Jr, Wiggins WF, Pomerantz SR, Andriole KP. Effects of age and sex on the distribution and symmetry of lumbar spinal and neural foraminal stenosis: a natural language processing analysis of 43,255 lumbar MRI reports. Neuroradiology. 2021 Jun;63(6):959–966.	17. Caton PW, Choudhury M, Hart AR, Cooke J, Belli AM, McNally DS. Sex differences in the severity of lumbar spinal stenosis on imaging: a systematic review and meta-analysis. Neuroradiology. 2021;63(2):189–99.
18. Peteler R, Schmitz P, Loher M, Jansen P, Grifka J, Benditz A. Sex-Dependent Differences in Symptom-Related Disability Due to Lumbar Spinal Stenosis. J Pain Res. 2021 Mar 16;14:747–755.	18. Peteler A, Lurie J, Tosteson T, Zhao W, Morgan TS, Tosteson AN. Sex differences in the severity of symptoms and functional limitations among patients with lumbar spinal stenosis. J Pain Res. 2021;14:123–31.
19. Kreiner DS, Shaffer WO, Baisden JL, Gilbert TJ, Summers JT, Toton JF, Hwang SW, Mendel RC, Reitman CA. An evidence-based clinical guideline for the diagnosis and treatment of degenerative lumbar spinal stenosis (update). Spine J 2013 Jul 1;13(7):734 − 43	19. Kreiner DS, Shaffer WO, Baisden JL, Gilbert TJ, Summers JT, Toton JF, et al. An evidence-based clinical guideline for the diagnosis and treatment of degenerative lumbar spinal stenosis (update). Spine J. 2013;13(7):734–43.
20. Wessberg P, Frennered K. Central lumbar spinal stenosis: natural history of non-surgical patients. Eur Spine J. 2017 Oct;26(10):2536-42.	20. Wessberg P, Frennered K. Central lumbar spinal stenosis: natural history and long-term outcome. Spine (Phila Pa 1976). 2017;42(7):E351–8.
21. Kobayashi S. Pathophysiology, diagnosis and treatment of intermittent claudication in patients with lumbar canal stenosis. World J Orthop. 2014 Apr 18;5(2):134.	21. Kobayashi S. Pathophysiology, diagnosis and treatment of intermittent claudication in patients with lumbar Canal stenosis. World J Orthop. 2014;5(2):134–45.
22. Ammendolia C, Côté P, Southerst D, Schneider M, Budgell B, Bombardier C, Hawker G, Rampersaud YR. Comprehensive nonsurgical treatment versus self-directed care to improve walking ability in lumbar spinal stenosis: a randomized trial. Arch Phys Med Rehabil. 2018 Dec 1;99(12):2408-19.	22. Ammendolia C, Stuber KJ, Tomkins-Lane C, Schneider M, Rampersaud YR, Furlan AD, et al. What interventions improve walking ability in neurogenic claudication with lumbar spinal stenosis? A systematic review. Eur Spine J. 2014;23(6):1282–301.
23. Imagama S, Matsuyama Y, Sakai Y, Ito Z, Wakao N, Deguchi M, Hachiya Y, Osawa Y, Yoshihara H, Kamiya M, Kanemura T. An arterial pulse examination is not sufficient for diagnosis of peripheral arterial disease in lumbar spinal canal stenosis: a prospective multicenter study. Spine. 2011 Jul 1;36(15):1204-10.	23. Imagama S, Matsuyama Y, Sakai Y, Ito Z, Wakao N, Hirano K, et al. Relationship between vascular disease and lumbar spinal stenosis: a prospective multicenter study. Spine (Phila Pa 1976). 2011;36(10):E703–8.
24. Collins TC, Johnson M, Henderson W, Khuri SF, Daley J. Lower extremity nontraumatic amputation among veterans with peripheral arterial disease: is race an independent factor?. Med Care. 2002 Jan 1;40(1):I-106.	24. Collins TC, Johnson M, Henderson W, Khuri SF, Daley J. Lower extremity nontraumatic amputation among veterans with peripheral arterial disease: is race an independent factor? Med Care. 2002;40(1):I106–16.
25. Botwin K, Brown LA, Fishman M, Rao S. Fluoroscopically guided caudal epidural steroid injections in degenerative lumbar spine stenosis. Pain Physician. 2007;10(4):547.	25. Botwin KP, Gruber RD, Bouchlas CG, Torres-Ramos FM, Sanelli JT, Freeman ED, et al. Degenerative lumbar spinal stenosis: a prospective study of epidural steroid injection and facet joint injection. Pain Physician. 2002;5(3):282–8.
26. Devin CJ, McCullough KA, Morris BJ, Yates AJ, Kang JD. Hip-spine syndrome. J Am Acad Orthop Surg. 2012 Jul 1;20(7):434 − 42.	26. Devin CJ, McGirt MJ. Best evidence in multimodal pain management in spine surgery and means of assessing postoperative pain and functional outcomes. J Clin Neurosci. 2012;19(9):1130–5.
27. Williams BS, Cohen SP. Greater trochanteric pain syndrome: a review of anatomy, diagnosis and treatment. Anesth Analg. 2009 May 1;108(5):1662-70.	27. Williams BS, Cohen SP. Greater trochanteric pain syndrome: a review of anatomy, diagnosis and treatment. Anesth Analg. 2009;108(5):1662–70.
28. Smith AY, Woodside JR. Urodynamic evaluation of patients with spinal stenosis. Urology. 1988 Nov 1;32(5):474-7.	28. Smith AY, Fuentes JM, Pierce MW, O’Connor JP. Lumbar spinal stenosis and its effect on sexual function: a review. Urology. 1988;32(1):1–6.
29. Nichols NM, Yerneni K, Chiu AB, Lu AY, Tan LA. Priapism associated with lumbar stenosis: case report and literature review. J Spine Surg. 2019 Dec;5(4):596.	29. Nichols P, Popham P, Kothari MJ. Neurogenic claudication and intermittent priapism: a case report. J Spinal Disord Tech. 2019;32(2):E91–3.
30. Santananukarn M, Pasutharnchat N. Isolated intermittent neurogenic priapism: an unusual presentation in degenerative lumbar spinal stenosis. BMJ Case Rep. 2019 Apr 1;12(4):e228107.	30. Santanananukarn M, Chotivichit A, Chotivichit A. Intermittent priapism in a patient with lumbar spinal stenosis: a case report. J Med Case Rep. 2019;13(1):1–4.
31. Rojas JI, Zurrú-Ganen MC, Romano M, Patrucco L, Cristiano E. Intermittent priapism as a clinical presentation of the lumbar narrow canal. Rev. neurol. (Ed. impr.). 2007:532–534.	31. Rahimizadeh A, Rahimizadeh S, Soufiani H, Rahimizadeh A. Intermittent priapism in a patient with lumbar spinal stenosis: a case report. J Med Case Rep. 2019;13(1):1–4.
32. Kanala DL. Intermittent priapism in degenerative lumbar spinal stenosis: case report. Turk Neurosurg. 2007;17(4):260-3	32. Hirota Y, Kawai M, Yamada K, Kawai M. Intermittent priapism in a patient with lumbar spinal stenosis: a case report. J Med Case Rep. 2020;14(1):1–4.
33. Gempt J, Rothoerl RD, Grams A, Meyer B, Ringel F. Effect of lumbar spinal stenosis and surgical decompression on erectile function. Spine. 2010 Oct 15;35(22):E1172-7.	33. Gempt J, Buchmann N, Lehmberg J, Meyer B. Erectile dysfunction in patients with lumbar spinal stenosis. J Neurosurg Spine. 2010;13(5):552–8.
34. Wottrich S, Kha S, Thompson N, Bakar D, Yee P, Melillo A, Nash C, Healy AT, Steinmetz M, Mroz T. The effect of cervical and lumbar decompression surgery for spinal stenosis on erectile dysfunction. Global Spine J. 2024 May;14(4):1193–1200.	34. Wotrich T, Schmid M, Schmid M, Schmid M. Erectile dysfunction in patients with lumbar spinal stenosis: a case series. J Neurosurg Spine. 2022;36(1):1–5.
35. Bussières A, Cancelliere C, Ammendolia C, Comer CM, Al Zoubi F, Châtillon CE, Chernish G, Cox JM, Gliedt JA, Haskett D, Jensen RK. Non-surgical interventions for lumbar spinal stenosis leading to neurogenic claudication: a clinical practice guideline. J Pain. 2021 Sep 1;22(9):1015–1039.	35. Bussieres AE, Stewart G, Al-Zoubi F, Decina P, Descarreaux M, Haskett D, et al. Spinal manipulative therapy for the management of low back pain: a guideline from the Canadian chiropractic guideline initiative. J Manipulative Physiol Ther. 2018;41(4):265–93.
36. Kirker K, Masaracchio MF, Loghmani P, Torres-Panchame RE, Mattia M, States R. Management of lumbar spinal stenosis: a systematic review and meta-analysis of rehabilitation, surgical, injection, and medication interventions. Physiother Theory Pract. 2023 Feb 1;39(2):241 − 86.	36. Comer CM, Redmond AC, Bird HA, Conaghan PG, Tennant A. Foot pain in rheumatoid arthritis: a comprehensive review. Foot (Edinb). 2022;52:101–8.
37. Martínez T, Mariscal G, de la Rubia Ortí JE, Barrios C. Efficacy and safety of pregabalin and gabapentin in spinal stenosis: a systematic review and meta-analysis. Front Pharmacol. 2023 Nov 29;14:1249478.	37. Martinez V, Attal N, Bouhassira D, Baudic S, Fletcher D. Systematic review and meta-analysis of the efficacy and safety of Pregabalin and Gabapentin in the treatment of neuropathic pain. Front Pharmacol. 2023;14:1–15.
38. Yaksi A, Ozgonenel L, Ozgonenel B. The efficiency of Gabapentin therapy in patients with lumbar spinal stenosis. Spine. (Phila Pa 1976). 2007;32(9):939–42.	38. Yaksi A, Ozgonenel L, Ozgonenel B. The efficiency of Gabapentin therapy in patients with lumbar spinal stenosis. Spine (Phila Pa 1976). 2007;32(9):939–42.
39. Sekiguchi M, Kikuchi SI. Efficacy of pregabalin in patients with sciatica: a randomized, double-blind, placebo-controlled trial. AME Med J. 2017 Jul 1;2(7).	39. Takahashi N, Kikuchi S, Shimizu T, Konno S. Gabapentin for the treatment of sciatica in patients with lumbar spinal stenosis: a randomized, double-blind, placebo-controlled trial. J Orthop Sci. 2014;19(4):600–4.
40. Markman JD, Jensen TS, Semel D, Li C, Parsons B, Behar R, Sadosky AB. Effects of pregabalin in patients with neuropathic pain previously treated with gabapentin: a pooled analysis of parallel-group, randomized, placebo‐controlled clinical trials. Pain Pract. 2017 Jul;17(6):718 − 28.	40. Markman JD, Frazer ME, Rast SA, Kennedy DJ, Manchikanti L. Gabapentin for chronic neuropathic pain: a randomized, double-blind, placebo-controlled trial. Pain Physician. 2015;18(4):E333–43.
41. Enke O, New HA, New CH, Mathieson S, McLachlan AJ, Latimer J, Maher CG, Lin CW. Anticonvulsants in the treatment of low back pain and lumbar radicular pain: a systematic review and meta-analysis. CMAJ. 2018 Jul 3;190(26):E786-93.	41. Enke O, New HA, New CH, Mathieson S, McLachlan AJ, Latimer J, et al. Anticonvulsants in the treatment of low back pain and lumbar radicular pain: a systematic review and meta-analysis. CMAJ. 2018;190(26):E786–93.
42. Shanthanna H, Gilron I, Rajarathinam M, AlAmri R, Kamath S, Thabane L, Devereaux PJ, Bhandari M. Benefits and safety of gabapentinoids in chronic low back pain: a systematic review and meta-analysis of randomized controlled trials. PLoS Med. 2017 Aug 15;14(8):e1002369.	42. Shanthanna H, Busse JW, Wang L, Kaushal A, Couban R, Riva JJ, et al. Gabapentinoids for chronic low back pain with and without radiculopathy: a systematic review and meta-analysis. PLoS Med. 2017;14(8):e1002369.
43. Qaseem A, Wilt TJ, McLean RM, Forciea MA, Clinical Guidelines Committee of the American College of Physicians*. Noninvasive treatments for acute, subacute, and chronic low back pain: a clinical practice guideline from the American College of Physicians. Ann Intern Med. 2017 Apr 4;166(7):514 − 30.	43. Qaseem A, Wilt TJ, McLean RM, Forciea MA. Noninvasive treatments for acute, subacute, and chronic low back pain: a clinical practice guideline from the American college of physicians. Ann Intern Med. 2017;166(7):514–30.
44. Cashin AG, Wand BM, O’Connell NE, Lee H, Rizzo RR, Bagg MK, O’Hagan E, Maher CG, Furlan AD, van Tulder MW, McAuley JH. Pharmacological treatments for low back pain in adults: an overview of Cochrane Reviews. Cochrane Database Syst Rev. 2023(4).	44. Cashin AG, Folly T, Bagg MK, Wewege MA, Jones MD, Booth J, et al. Acetaminophen for low back pain. Cochrane Database Syst Rev. 2023;1:CD014341.
45. Friedly JL, Comstock BA, Turner JA, Heagerty PJ, Deyo RA, Sullivan SD, Bauer Z, Bresnahan BW, Avins AL, Nedeljkovic SS, Nerenz DR. A randomized trial of epidural glucocorticoid injections for spinal stenosis. N Engl J Med. 2014 Jul 3;371(1):11–21.	45. Friedly JL, Bresnahan BW, Comstock BA, Turner JA, Deyo RA, Sullivan SD, et al. Systematic review of the effectiveness of lumbar epidural steroid injections in the management of chronic low back pain. Spine (Phila Pa 1976). 2007;32(24):E713–27.
46. Chou R, Hashimoto R, Friedly J, Fu R, Bougatsos C, Dana T, Sullivan SD, Jarvik J. Epidural corticosteroid injections for radiculopathy and spinal stenosis: a systematic review and meta-analysis. Ann Intern Med. 2015 Sep 1;163(5):373 − 81.	46. Chow R, Chiodo AE, Otis J, Bhatia A, Rapoport A, Coyle D, et al. Epidural corticosteroid injections for radiculopathy and spinal stenosis: a systematic review and meta-analysis. Ann Intern Med. 2015;163(5):373–81.
47. Kim KH, Kim TH, Lee BR, Kim JK, Son DW, Lee SW, Yang GY. Acupuncture for lumbar spinal stenosis: a systematic review and meta-analysis. Complement Ther Med. 2013 Oct 1;21(5):535 − 56.	47. Kwon CY, Lee B, Kim JW, Lee JH, Lee JH. Acupuncture for lumbar spinal stenosis: a systematic review and meta-analysis. Med (Baltim). 2019;98(26):e16208.
48. Passmore SR, Johnson MG, Aloraini SM, Cooper S, Aziz M, Glazebrook CM. Impact of spinal manipulation on lower extremity motor control in lumbar spinal stenosis patients: a small-scale assessor-blind randomized clinical trial. J Manipulative Physiol Ther. 2019 Jan 1;42(1):23–33.	48. Passmore SR, Bruno P, Toth A, Vernon H. Spinal manipulation for lumbar spinal stenosis-related symptoms: a randomized clinical trial. J Manipulative Physiol Ther. 2019;42(5):319–29.
49. Trager RJ, Baumann A, Baumann AN. Improvement of anorgasmia and anejaculation after spinal manipulation in an older man with lumbar stenosis: A case report. Cureus. 2023 Feb 7;15(2).	49. Trager RJ, Bauman A. Improvement of anorgasmia and anejaculation after spinal manipulation in an older man with lumbar spinal stenosis: a case report. Cureus. 2023;15(1):e12567.
50. Machado GC, Ferreira PH, Harris IA, Pinheiro MB, Koes BW, Van Tulder M, Rzewuska M, Maher CG, Ferreira ML. Effectiveness of surgery for lumbar spinal stenosis: a systematic review and meta-analysis. PloS one. 2015 Mar 30;10(3):e0122800.	50. Machado GC, Ferreira PH, Harris IA, Pinheiro MB, Koes BW, van Tulder M, et al. Effectiveness of surgery for lumbar spinal stenosis: a systematic review and meta-analysis. PLoS ONE. 2016;11(3):e0149986.
51. Katz JN, Harris MB. Lumbar spinal stenosis. N Engl J Med. 2008 Feb 21;358(8):818 − 25.	51. Katz JN, Harris MB. Clinical practice. Lumbar spinal stenosis. N Engl J Med. 2008;358(8):818–25.
52. Schneider MJ, Ammendolia C, Murphy DR, Glick RM, Hile E, Tudorascu DL, Morton SC, Smith C, Patterson CG, Piva SR. Comparative clinical effectiveness of nonsurgical treatment methods in patients with lumbar spinal stenosis: a randomized clinical trial. JAMA Netw Open. 2019 Jan 4;2(1):e186828	52. Schneider M, Ammendolia C, Murphy DR, Glick RM, Stevans JM, Schneider MJ, et al. Comparative effectiveness of a 6-week boot camp program versus usual physical therapy for lumbar spinal stenosis: a randomized controlled trial. J Pain Res. 2019;12:1–12.
53. Minetama M, Kawakami M, Teraguchi M, Kagotani R, Mera Y, Sumiya T, Nakagawa M, Yamamoto Y, Matsuo S, Koike Y, Sakon N. Supervised physical therapy vs. home exercise for patients with lumbar spinal stenosis: a randomized controlled trial. Spine J. 2019 Aug 1;19(8):1310-8.	53. Minatama M, Kawakami M, Teraguchi M, Kagotani R, Mera Y, Yamamoto I, et al. Effectiveness of a 6-week boot camp program for lumbar spinal stenosis: a randomized controlled trial. Spine (Phila Pa 1976). 2019;44(17):E1024–32.
54. Minetama M, Kawakami M, Teraguchi M, Kagotani R, Mera Y, Sumiya T, Nakagawa M, Yamamoto Y, Matsuo S, Sakon N, Nakatani T. Supervised physical therapy versus unsupervised exercise for patients with lumbar spinal stenosis: 1-year follow-up of a randomized controlled trial. Clin Rehabil. 2021 Jul;35(7):964 − 75.	54. Minatama M, Kawakami M, Teraguchi M, Kagotani R, Mera Y, Yamamoto I, et al. Long-term effectiveness of a 6-week boot camp program for lumbar spinal stenosis: a randomized controlled trial. Spine (Phila Pa 1976). 2022;47(1):E1–8.
55. Linton SJ, Boersma K. Early identification of patients at risk of developing a persistent back problem: the predictive validity of the Örebro Musculoskeletal Pain Questionnaire. Clin J Pain. 2003 Mar 1;19(2):80 − 6.	55. Linton SJ, Boersma K. Early identification of patients at risk of developing a persistent back problem: the predictive validity of the Örebro musculoskeletal pain questionnaire. Clin J Pain. 2003;19(2):80–6.
56. Simmonds MJ, Kumar S, Lechelt E. Psychosocial factors in disabling low back pain: causes or consequences?. Disabil Rehabil. 1996 Jan 1;18(4):161-8	56. Simmonds MJ, Kumar S, Lechelt E. Psychosocial factors in disabling low back pain: causes or consequences? Disabil Rehabil. 1996;18(4):161–8.
57. Anderson DB, Mobbs RJ, Smith ZA, De Luca K, Sabet T, Van Gelder JM. Importance of valid, reliable, and responsive outcome measures for lumbar spinal stenosis. Spine J. 2023 Mar 1;23(3):345-9.	57. Anderson DB, Mobbs RJ, Smith ZA, De Luca K, Sabet T, Van Gelder JM. Importance of valid, reliable, and responsive outcome measures for lumbar spinal stenosis. Spine J. 2023;23(3):345–9. Epub 2022 Nov 25. PMID: 36,436,767.
58. Howick J, Friedemann C, Tsakok M, Watson R, Tsakok T, Thomas J, Perera R, Fleming S, Heneghan C. Are treatments more effective than placebos? A systematic review and meta-analysis. PloS one. 2013 May 15;8(5):e62599	58. Howick J, Webster R, Rees JL, Turner R, Macdonald H, Price A, et al. Are treatments more effective than placebos? A systematic review and meta-analysis. PLoS ONE. 2013;8(5):e62599.
59. Rossettini G, Carlino E, Testa M. Clinical relevance of contextual factors as triggers of placebo and nocebo effects in musculoskeletal pain. BMC Musculoskelet Disord. 2018 Jan 22;19(1):27.	59. Rossettini G, Carlino E, Testa M. Clinical relevance of contextual factors as triggers of placebo and Nocebo effects in musculoskeletal pain. Front Psychol. 2019;10:1–12.
60. Ammendolia C, Plener J, Ahmed A, Hofkichner C. Enhancing positive contextual factors to improve outcomes in lumbar spinal stenosis: a conceptual framework. Poster presentation. 17th World Federation of Chiropractic Biennial Congress, Gold Coast, Australia; October 11–14, 2023.	60. Ammendolia C, Plener J, Ahmed A, Hofkichner C. Enhancing positive contextual factors to improve outcomes in lumbar spinal stenosis: a conceptual framework. Poster presentation. World Federation of Chiropractic Congress, Gold Coast, Australia; 2024. p. 10.
61. Sobański D, Staszkiewicz R, Gadzieliński M, Stachura MK, Czepko RA, Holiński M, Czepko R, Grabarek BO. A study of 179 patients with degenerative stenosis of the lumbosacral spine to evaluate differences in quality of life and disability outcomes at 12 months, between conservative treatment and surgical decompression. Med Sci Monit. 2023 May 22;29:e940213-1.	61. Sobański D, Staszkiewicz R, Gadzieliński M, Stachura MK, Czepko RA, Holiński M, Czepko R, Garbarek BO. A study of 179 patients with degenerative stenosis of the lumbosacral spine to evaluate differences in quality of life and disability outcomes at 12 months, between Conservative treatment and surgical decompression. Med Sci Monit. 2023;29:e940213.
62. Ammendolia C. Degenerative lumbar spinal stenosis and its imposters: three case studies. J Can Chiropr Assoc. 2014 Sep;58(3):312-9.	Originally not included

